# Carcinoma in the Negro

**Published:** 1897-12

**Authors:** J. Collins Warren

**Affiliations:** Boston


### Correspondence.

### CARCINOMA IN THE NEGRO.

Boston, October 25, 1897.

58 Beacon St.

*To the Editor :*

Sir :—In your valuable journal for October I find on page 174 that Dr. W. F. Westmoreland states in the *American Journal of Surgery and Gynecology* "that Dr. Warren's declaration (in his text-book of Surgical Pathology, 1895) 'that carcinoma does not occur in the negro is wholly incorrect.' He has seen at least a half dozen cases in the last two years." I find that on page 643 of my Surgical Pathology, in speaking of the geographical distribution of cancer, I say : "Negroes are generally supposed in America to be much less afflicted with cancer than the white race." Any facts bearing upon this subject are of great interest and I should be glad to hear a further expression of opinion from Southern surgeons. I have never seen a case of cancer of the breast in the negress and should be glad to be informed in regard to any case. I have, however, had under my care a very malignant form of cancer of the uterus in a full-blooded negress. Respectfully yours,

J. COLLINS WARREN.

